# Strychnine

**Published:** 1854-12

**Authors:** 


Strychnine.—Much discussion has arisen in Paris with respect to this remedy. M. Abeille, its advocate, published a most sanguine statement of its powers, and termed it as certain a specific in cholera as quinine in ague. He gave the sulphate of strychnine in doses of one-third of a grain in two ounces of water four times in each twenty-four hours; at the same periods he applied thirty or forty leeches on the base of the thorax, according to the strength of the patient. The statements made by G. M. Abeille, have led to the employment of strychnine by many physicians, viz: See, Grisolle, Renouard, Fremy, Herard, and Vernois, and the result has been that not one of those gentlemen, after a very great experience of strychnine, has been able to perceive the least benefit in bad cases. In slight cases See thinks it useful. Moreover, it is stated by the Editor of the *Bull. Gén. de Thér.* (No. 4, 30 Aout, p. 199), that he himself went to the *Hôpital du Roule*, where M. Abeille practices, and found that no other of the physicians there, including M. Boudin, in whose charge the cholera patients were, had the least faith in the practice. The report has also been read to the Academie (L'Union, Sept. 6,) by M. Gerardin, on the documents submitted by M. Abeille, in which the statements of this gentleman are shown to be without support, even from his own evidence. We may observe that strychnine has been tried before, and found wanting.—*Bull. Gén. de Thér.*

